# Predicting graft failure in pediatric liver transplantation based on early biomarkers using machine learning models

**DOI:** 10.1038/s41598-022-25900-0

**Published:** 2022-12-27

**Authors:** Seungho Jung, Kyemyung Park, Kyong Ihn, Seon Ju Kim, Myoung Soo Kim, Dongwoo Chae, Bon-Nyeo Koo

**Affiliations:** 1 Department of Anesthesiology and Pain Medicine, Anesthesia and Pain Research Institute, Yonsei University College of Medicine, 50-1 Yonsei-ro, Seodaemun-gu, Seoul, 03722 Republic of Korea; 2 Department of Biomedical Engineering, College of Information and Biotechnology, Ulsan National Institute of Science and Technology (UNIST), 50, UNIST-gil, Ulju-gun, Ulsan, 44919 Republic of Korea; 3 Department of Surgery, Yonsei University College of Medicine, Seoul, Republic of Korea; 4 Department of Anesthesiology and Pain Medicine, Yongin Severance Hospital, Yonsei University College of Medicine, Yongin, Republic of Korea; 5 Department of Pharmacology, Yonsei University College of Medicine, 50-1 Yonseiro, Seodaemun-gu, Seoul, 03722 Republic of Korea

**Keywords:** Machine learning, Paediatric research, Risk factors, Hepatology, Prognosis

## Abstract

The early detection of graft failure in pediatric liver transplantation is crucial for appropriate intervention. Graft failure is associated with numerous perioperative risk factors. This study aimed to develop an individualized predictive model for 90-days graft failure in pediatric liver transplantation using machine learning methods. We conducted a single-center retrospective cohort study. A total of 87 liver transplantation cases performed in patients aged < 12 years at the Severance Hospital between January 2010 and September 2020 were included as data samples. Preoperative conditions of recipients and donors, intraoperative care, postoperative serial laboratory parameters, and events observed within seven days of surgery were collected as features. A least absolute shrinkage and selection operator (LASSO) -based method was used for feature selection to overcome the high dimensionality and collinearity of variables. Among 146 features, four variables were selected as the resultant features, namely, preoperative hepatic encephalopathy, sodium level at the end of surgery, hepatic artery thrombosis, and total bilirubin level on postoperative day 7. These features were selected from different times and represent distinct clinical aspects. The model with logistic regression demonstrated the best prediction performance among various machine learning methods tested (area under the receiver operating characteristic curve (AUROC) = 0.898 and area under the precision–recall curve (AUPR) = 0.882). The risk scoring system developed based on the logistic regression model showed an AUROC of 0.910 and an AUPR of 0.830. Together, the prediction of graft failure in pediatric liver transplantation using the proposed machine learning model exhibited superior discrimination power and, therefore, can provide valuable information to clinicians for their decision making during the postoperative management of the patients.

## Introduction

The number of pediatric patients who receive liver transplant has increased over the years^1^. Liver transplantation is the standard treatment for children with end-stage liver disease, malignancy, and metabolic disorders related to liver^2^. However, the number of suitable donor livers is limited and numerous children remain on the waiting list for long periods or even die before receiving a transplant^3^. Owing to better management during the pre-transplant and post-transplant phases, the graft survival rate has improved^4^. However, the individualized prediction of graft failure in pediatric liver transplantation remains challenging because there are numerous different perioperative factors including the preoperative status of patients, intraoperative anesthetic management and postoperative complications such as vascular thrombosis or biliary leakage that can together influence the outcome of transplantation^2,5,6^. Moreover, the number of pediatric liver transplant patients in single centers is usually small. The use of traditional statistical methods can be limited under these conditions.

Several studies have employed statistical methods to evaluate the predictive factors of graft failure and developed predictive models for pediatric liver transplantation^7–10^. However, the complex interactions between numerous parameters derived from patient medical records make it difficult to select specific predictors of graft failure. Recently, several attempts have been made to analyze medical data using machine learning for application in clinical practice^11,12^. Several machine learning models have been used to predict graft survival/failure in liver transplantation (Supplementary Table S1)^13–15^. However, pediatric patients are excluded, and serial early biomarkers are not used as features in those models.

Machine learning techniques enable the analysis of considerable amounts of complex data and the development of models for estimating risks or predicting events. Specifically, the least absolute shrinkage and selection operator (LASSO) -based method can be used in the case of relatively many collected features in comparison to the subjects, and high correlation among these features^16,17^. By using this method, we tried to overcome the high correlation of features such as serial laboratory data which were collected from a small number of patients.

The objective of this study involved the development of a high-performance machine learning model for individualized prediction of 90-day graft failure in pediatric liver transplantation using perioperative parameters collected within 7 days after surgery. For this, we collected patient data from the electronic medical records at a single center retrospectively. The dataset included 146 features that exceed the number of patients, thereby intractable when using traditional statistical methods. We utilized a LASSO-based method to identify the potential predictors of 90-day graft failure. These predictors served as input variables for the development of machine learning models. Graft failure was defined as failure of the liver allograft that required re-transplantation or resulted in death. Deaths caused by other than liver failure were not defined as graft failure. Finally, we developed a risk scoring system of graft failure in patients for easy utilization in the routine clinical setting.

## Methods

### Patients and data collection

This study was approved by Institutional Review Board (IRB) of our hospital (IRB No. 4–2018-0205). The requirement for informed consent was waived by the IRB of Severance Hospital, Yonsei University Health System, Seoul, Korea, owing to the retrospective nature of this study. All methods were performed in accordance with the relevant guidelines and regulations. We conducted a single-center retrospective cohort study. The data were obtained from electronic medical records. Patients aged below 12 years who underwent pediatric liver transplantation surgeries at the Severance Hospital between January 2010 and September 2020 were selected as the subjects.

Moreover, the characteristics of recipients and donors, anesthetic and surgical events, complications during hospitalization, and serial laboratory results until the seventh postoperative day (Supplementary Table S2) were collected as the features. The primary endpoint was graft failure at 90 days after transplantation.

### Statistical and machine learning methods

#### Nested cross-validation

**Figure 1:**
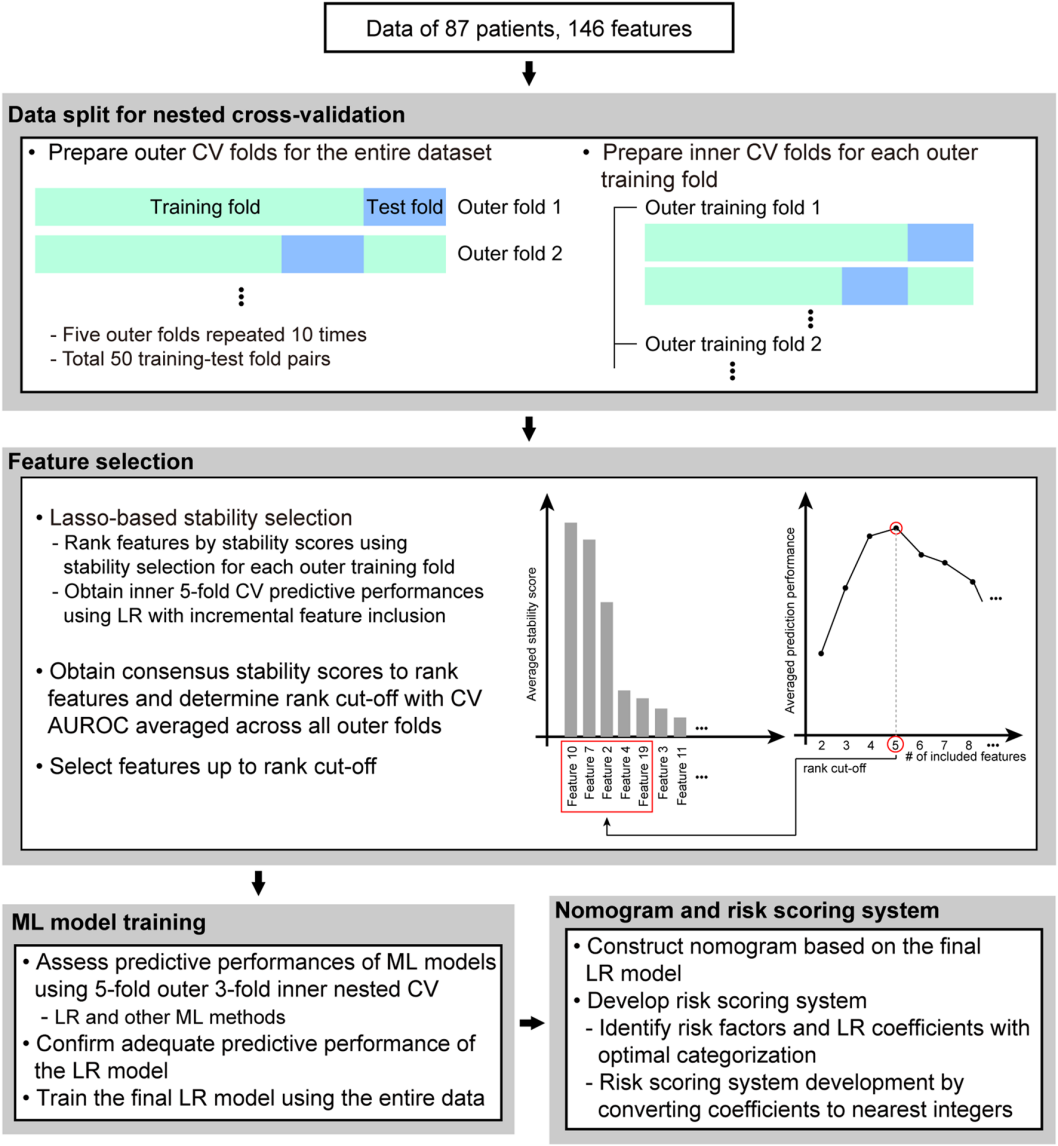
Schematic of the overall workflow. *CV* cross-validation, *LR* Logistic regression, *ML* machine learning, *AUROC* area under receiver operation characteristic curve.

Because the total number of transplantations and graft failure cases was small, splitting the dataset into training and testing could cause additional uncertainties in the analysis. Therefore, we employed a nested cross-validation scheme for feature selection and predictive machine-learning model development. The training–test split was repeated as outer cross-validation (Fig. 1). For each outer training fold, additional inner cross-validation was applied for feature selection, hyperparameter tuning, and performance assessment during model development.

#### Feature selection

To select the features, we employed stability selection, a LASSO-based method implemented in the R package (*stabs*)^18^. This method assesses how often (or stably) each feature is selected by LASSO across bootstrapped subsamples for the given input data by quantifying the stability score (the number of inclusions/bootstrapped subsamples) of each feature. Bootstrapping ensures robustness and the effective reduction of selecting false positives. The features listed in Table 1 and Supplementary Table S2 were considered candidate predictors. Given the missing values (119 out of 12,702 items) across subjects (17 out of 87 subjects) and features (56 out of 146 features) and high collinearity between features, we sought to maximize the utilization of data in feature selection by filling the missing values with the mean values of the corresponding features. Stability selection was applied with five-fold outer and inner nested cross-validation, repeated 10 times (Fig. 1). For each outer training fold, the features were ranked in the order of their decreasing stability scores. Logistic regression models with incrementally added top-ranking features were then evaluated based on the predictive performance of five-fold inner cross-validation. After all iterations across the outer folds, the consensus stability scores for all features were obtained by averaging the stability scores across the outer folds. Subsequently, the optimal number of features was determined as the minimum number of features associated with the maximum averaged cross-validated predictive performance. Features were then selected up to the rank of the optimal number in the consensus stability scores, preoperative hepatic encephalopathy (HE), Na level at the end of operation (Endop_Na), hepatic artery thrombosis (HA_thrombosis), and total bilirubin level on postoperative day (POD) 7 (POD7_Tbilirubin).

**Table 1:** Demographics and perioperative state.

	All	No graft failure	Graft failure	*p* value
	N = 87	N = 70 (80.5%)	N = 17 (19.5%)	
**Recipient**				
Age (month)	36.8 (± 40.5)	36.0 (± 40.3)	39.9 (± 42.3)	0.716
Female	52 (59.8%)	41 (58.6%)	11 (64.7%)	0.785
Intrauterine period (week)	38.4 (± 2.0)	38.3 (± 2.2)	39.1 (± 1.1)	0.224
Normal spontaneous vaginal delivery	49 (56.3%)	40 (57.1%)	9 (52.9%)	0.790
Body weight (kg)	13.7 (± 8.5)	13.7 (± 8.5)	13.5 (± 8.8)	0.604
Body weight < 10 kg	47 (54.0%)	38 (54.3%)	9 (52.9%)	> 0.999
Reason of liver transplant surgery				0.0549
Biliary atresia	65 (74.7%)	56 (80.0%)	9 (52.9%)	
Acute liver failure	10 (11.5%)	6 (8.6%)	4 (23.5%)	
Metabolic liver disease	2 (2.3%)	1 (1.4%)	1 (5.9%)	
Hepatocellular carcinoma	3 (3.5%)	3 (4.3%)	0 (0%)	
Graft failure	1 (1.2%)	1 (1.4%)	0 (0%)	
Budd–Chiari syndrome	3 (3.5%)	2 (2.9%)	1 (5.9%)	
Cirrhosis, cryptogenic	3 (3.5%)	1 (1.4%)	2 (11.8%)	
Others	65 (74.7%)	56 (80.0%)	9 (52.9%)	
**Preop. conditions**				
Re-transplantation	47 (54.0%)	38 (54.3%)	9 (52.9%)	> 0.999
Pediatric end-stage liver disease score	11.1 (± 12.0)	10.8 (± 12.1)	12.5 (± 11.8)	0.668
Child–Pugh score	8.7 (± 2.0)	8.6 (± 2.0)	9.2 (± 2.4)	0.180
Previous operation histroy	66 (75.9%)	57 (81.4%)	9 (52.9%)	0.024
Ascites	49 (56.3%)	43 (61.4%)	6 (35.3%)	0.061
Hepatic encephalopathy	11 (12.6%)	4 (5.7%)	7 (41.2%)	< 0.001
Esophageal varix	45 (51.7%)	40 (57.1%)	5 (29.4%)	0.058
Splenomegaly	75 (86.2%)	64 (91.4%)	11 (64.7%)	0.011
Abnormal echocardiography	22 (25.3%)	18 (25.7%)	4 (23.5%)	> 0.999
Intensive care unit admission	25 (28.7%)	20 (28.6%)	5 (29.4%)	> 0.999
Mechanical ventilation	18 (20.7%)	14 (20.0%)	4 (23.5%)	0.745
ASA				0.782
2	1 (1.15%)	1 (1.43%)	0 (0%)	
3	14 (16.1%)	11 (15.7%)	3 (17.6%)	
4	69 (79.3%)	56 (80%)	13 (76.5%)	
5	3 (3.45%)	2 (2.86%)	1 (5.88%)	
**Surgery**				
Blood loss per estimated blood volume	1.3 (± 2.3)	1.1 (± 1.0)	2.1 (± 4.9)	0.864
Urinary output (mL/kg/h)	2.8 (± 2.0)	2.6 (± 1.3)	3.8 (± 3.5)	0.046
Operation time (h)	11.5 (± 2.3)	11.3 (± 2.3)	12.3 (± 2.4)	0.103
Anesthetic time (h)	12.9 (± 2.2)	12.7 (± 2.2)	13.7 (± 2.3)	0.127
Type				0.057
Elective	40 (46.0%)	36 (51.4%)	4 (23.5%)	
Emergency	47 (54.0%)	34 (48.6%)	13 (76.5%)	
Intraoperative vasopressor use	62 (71.3%)	49 (70.0%)	13 (76.5%)	0.768
Cold ischemic time (h)	3.6 (± 3.2)	3.3 (± 2.8)	4.9 (± 4.5)	0.413
Warm ischemic time (min)	58.6 (± 32.9)	59.0 (± 35.0)	56.94 (± 23.0)	0.851
Reperfusion time (h)	6.1 (± 1.5)	6.1 (± 1.5)	6.3 (± 1.8)	0.940
Anhepatic phase (h)	1.7 (± 0.8)	1.8 (± 0.8)	1.6 (± 0.6)	0.879
Perioperative CRRT	23 (26.4%)	14 (20.0%)	9 (52.9%)	0.012
Preoperative CRRT	10 (11.5%)	6 (8.6%)	4 (23.5%)	0.100
Intraoperative CRRT	13 (14.9%)	9 (12.9%)	4 (23.5%)	0.272
Postoperative CRRT	25 (28.7%)	15 (21.4%)	10 (58.8%)	0.005
**Surgery cx**				
Hepatic artery thrombosis	4 (4.6%)	0 (0%)	4 (23.5%)	0.001
Portal vein thrombosis	6 (6.9%)	2 (2.9%)	4 (23.5%)	0.012
Hepatic vein thrombosis	1 (1.2%)	1 (1.4%)	0 (0%)	> 0.999
Biliary duct complication				> 0.999
Bile leakage	7 (8.1%)	6 (8.6%)	1 (5.9%)	
Biliary stricture/obstruction	2 (2.3%)	2 (2.9%)	0 (0%)	
Infection	19 (21.8%)	16 (22.9%)	3 (17.6%)	0.754
Acute cellular rejection	9 (10.3%)	8 (11.4%)	1 (5.9%)	0.682
**Donor**				
Donor status				0.261
Living	57 (65.5%)	48 (68.6%)	9 (52.9%)	
Deceased	30 (34.5%)	22 (31.4%)	8 (47.1%)	
ABO mismatch	32 (36.8%)	27 (38.6%)	5 (29.4%)	0.582
Female	62 (71.3%)	52 (74.3%)	10 (58.8%)	0.238
Age (year)	29.3 (± 9.8)	30.0 (± 8.2)	26.7 (± 14.6)	0.987

Abbreviations: *ASA* American Society of Anesthesiology, *CRRT* Continuous renal replacement therapy.

The numbers denote the mean (SD) or number of patients (percentage).

The *p* values were obtained using Fisher’s exact test for categorical features and t-test (or Mann–Whitney test) for normally (or non-normally) distributed continuous features.

#### Machine learning methods

Based on the selected features, we constructed machine learning models using logistic regression, elastic net, random forests, extreme gradient boosting, support vector machines (SVM), and neural networks. We used the dataset without two patients with missing values under POD7_Tbilirubin, which obtained the second highest rank in the consensus stability scores. We employed five-fold outer and three-fold inner nested cross-validation, repeated 10 times, to assess and compare the model performance across machine learning methods and the hyperparameters of each machine learning method using the R *caret* package. For the prediction performance, we computed the area under the receiver operating characteristic (AUROC) and area under the precision–recall (AUPR) curves. A nomogram was constructed based on the final logistic regression model using the R *rms* package.

#### Risk scoring system

The risk scoring system was developed by first transforming the continuous features into categorical ones by binning. The regression coefficients of the categories were then used to assign appropriate risk scores. To determine the optimal binning boundaries, we first obtained the deciles of each continuous feature, generating nine binary categorization schemes, and retrained two-fold cross-validated logistic regression models for each categorization scheme. Subsequently, we selected the scheme with the highest cross-validated AUROC. The same procedure was applied recursively until the cross-validated AUROC did not improve to determine the optimal bins for all continuous features, which were then filtered based on the statistical significance of the regression coefficients of categorical features and their effects on the Akaike information criteria. Finally, the scores of categorical features were derived from regression coefficients by finding the corresponding integer values that best preserved the relative ratios between the coefficients.

#### Software

All analyses were performed using R (version 3.6.2).

## Results

Among the 87 cases of pediatric liver transplantations selected in this study, 17 eventually developed liver graft failure 90 days after the surgery (Table 1). The baseline demographic data, perioperative conditions, laboratory data, and complication-related features differed significantly (*p* < 0.05) across the graft outcomes (Table 1 and Supplementary Table S2). On POD 1, the transplanted liver with potential graft failure already showed signs of tissue destruction (reflected by higher ALT), followed by decreased function of producing the coagulation factor on POD 2 (reflected by higher aPTT) and decreased excretion function (reflected by higher total and direct bilirubin) on POD 7 (see Supplementary Table S3 and Supplementary Fig. S1). These significant individual associations between input features and graft outcomes suggested that early patient-derived features are likely to have predictive power for the graft status at later time points. However, the overall high dimensionality of data, with 146 input features greater than the number of patients, and high collinearity between redundant features, such as the repeatedly measured ones across multiple time points, presented challenges for distinguishing most predictive features to develop predictive models of liver graft failure (Supplementary Fig. S2).

**Figure 2:**
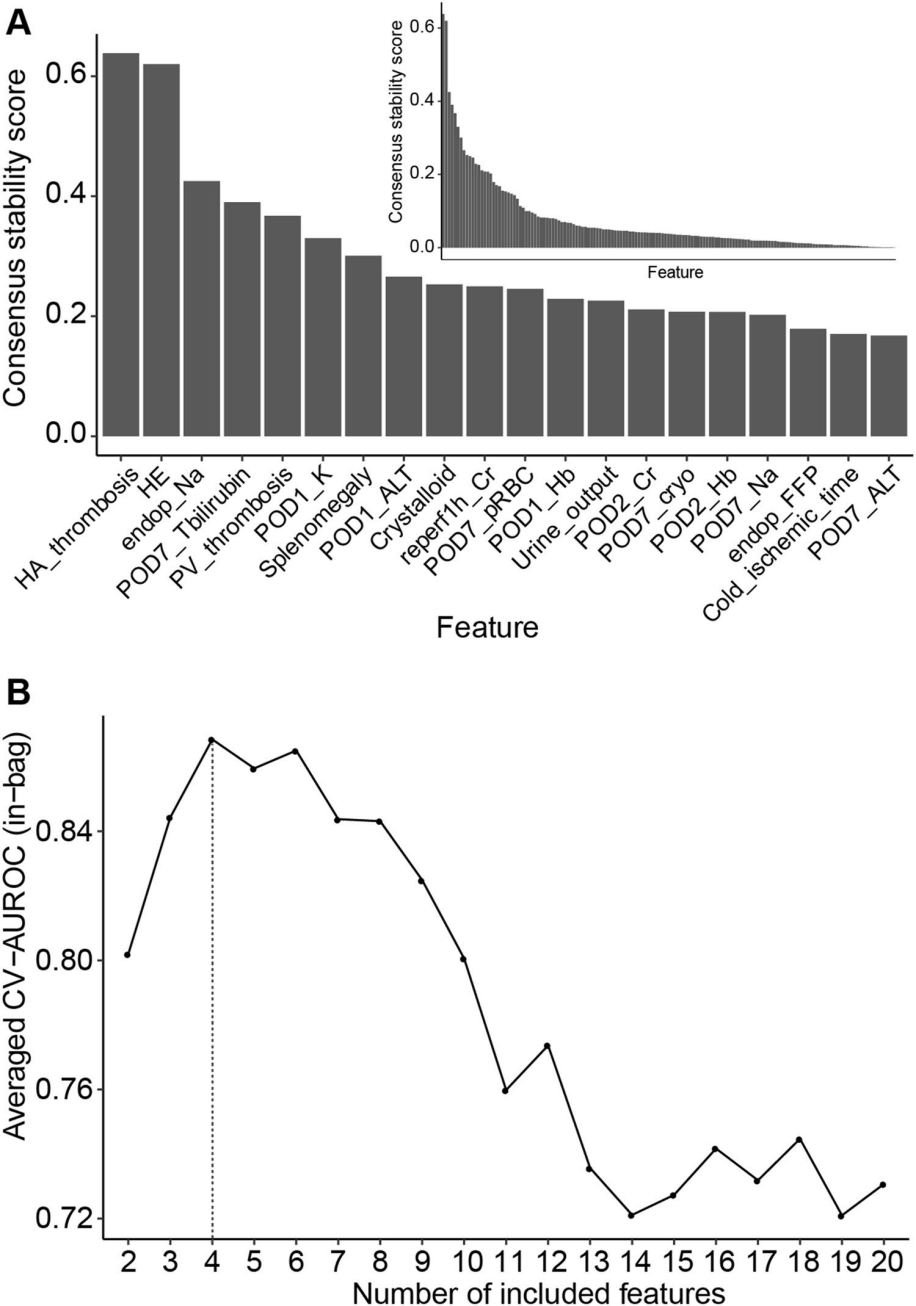
Feature selection results. (**A**) Features ordered by consensus stability scores averaged across outer cross-validation folds (Methods). (Outset) Top 20 features. Selected features based on the optimal number of features are in bold. (Inset) Overall distribution of stability scores among all features. (**B**) Averaged cross-validated AUROC with incremental numbers of features. The grey vertical line indicates the optimal number of features.

To address this, we conducted feature selection using stability selection, a LASSO-based method in the nested cross-validation scheme (Fig. 1). The consensus stability scores obtained by stability selection were ranked in decreasing order, as shown in Fig. 2A. The top four features were determined as the rank cutoff because this was the optimal number of features that resulted in the best averaged cross-validation AUROC curve across the outer training fold (Fig. 2B). The selected features included preoperative HE, Na level at the end of operation (Endop_Na), hepatic artery thrombosis (HA_thrombosis), and total bilirubin level on POD7 (POD7_Tbilirubin). Interestingly, these are from various time points and reflect various pathophysiological aspects of liver transplant surgery. This suggests that our feature selection approach delineated non-redundant predictive features, thereby overcoming the high dimensionality and collinearity of the dataset.

Next, we sought to build predictive machine learning models based on these selected features. Logistic regression is one of the best models, along with elastic net, based on the cross-validation performance (AUROC = 0.898 and AUPR = 0.882; Supplementary Table S4 and Supplementary Fig. S3). The final logistic regression models were generated using the entire dataset, as summarized in Supplementary Table 5.

**Figure 3:**
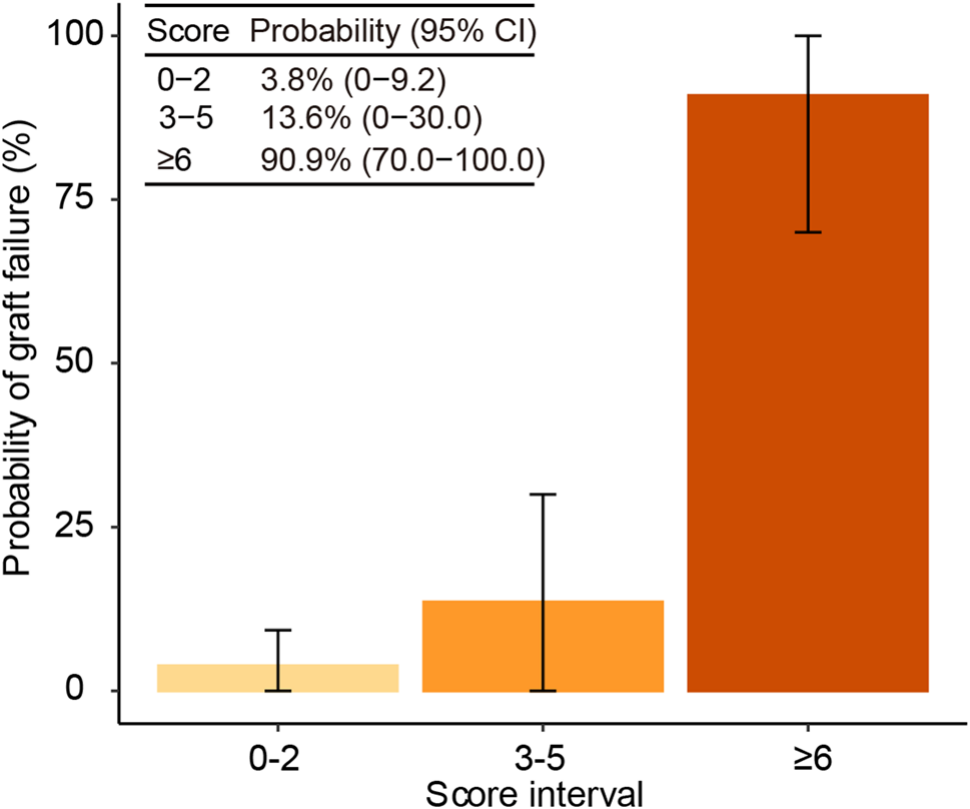
Probability of graft failure in score intervals based on the scoring system.

Finally, we constructed a nomogram based on the final logistic regression model (Supplementary Fig. S4). In addition, we developed a risk scoring system after categorizing the continuous features (Table 2 and Fig. 3). The cross-validation prediction performance of the optimal categorized logistic regression model exhibited an AUROC and AUPR of 0.910 and 0.830, respectively. The scoring system robustly reflected the categorized logistic regression model with a Pearson correlation coefficient of 1.00 between the scores and linear predictors. The scoring system could delineate a 50-fold difference in the risk of graft failure across score intervals. These findings may guide early therapeutic interventions to prevent graft failure after liver transplant surgeries.

**Table 2:** Risk scoring system.

Factor	Score
**HA_thrombosis**	
Yes	+ 27
**HE**	
Yes	+ 4
**POD7_Tbilirubin**	
$\ge$ 2 and $<$ 8	+ 2
$\ge$ 8	+ 5
**Endop_Na**	
$\ge$ 146	+ 3

Abbreviations: *HA_thrombosis* hepatic artery thrombosis, *HE* preoperative hepatic encephalopathy, *POD7_Tbilirubin* total bilirubin level on POD7, *Endop_Na* Na level at the end of operation, *POD* Post operative day.

## Discussion

We developed a predictive machine learning model for 90-days graft failure in pediatric liver transplantation patients using the features derived until POD 7. The number of extracted features was greater than the number of observed features. Therefore, we used a LASSO-based method to overcome the high dimensionality and collinearity of features. To further ensure robust feature selection by minimizing the detection of false positive features, we employed the stability selection method developed by Meinshausen and Buhlmann in nested cross-validation^18^. The model with logistic regression based on the selected features exhibited the best prediction performance (AUROC = 0.898 and AUPR = 0.882). Furthermore, we developed a risk scoring system to predict graft failure.

Pediatric liver transplantation differs from adult transplantation in terms of etiology and outcome^19^. Some models use traditional statistical approaches for predicting the prognosis of pediatric transplantation^8–10^. Recently, machine learning methods have exhibited outstanding performance in analyzing large volumes of medical data and predicting the outcomes of patients to help clinicians in decision making^20^. The early detection of graft failure is important for registration on the waiting list when patients need to undergo re-transplantation. In pediatric liver transplantation, specifically, the proportion of living donor transplantation is higher than that of deceased donor transplantation when compared to the same proportion in adult liver transplantation^3,21^. Therefore, the early prediction of graft failure is essential for the prompt evaluation of other living candidate donors.

Recently, Wadhwani et al. reported a machine learning model for predicting the ideal outcomes three years after pediatric liver transplantation^22^. They used features including the postoperative characteristics until one year after surgery. We aimed to predict earlier phase graft outcomes for surgeons to prepare for proper management or re-transplantation. According to a recent outcome analysis using the National Registry of Korea, the Kaplan–Meier analysis of survival rates of patients and grafts showed a rapidly decreasing curve until three months after transplantation, followed by flattening of the curve after three months. The graft survival rates reached a plateau area after three months^21^. This is why we predicted 90-days graft failure using only perioperative data collected until POD 7. We also used serial perioperative laboratory data for each patient to obtain more precise predictions and personalized algorithms.

HE is a brain dysfunction caused by liver insufficiency and/or portosystemic blood shunting^23^. The pathophysiology of HE has not been completely understood^24^. However, it has been reported as an independent risk factor of poor outcome in liver transplantation^25^. Recently, Sahinturk et al. reported that preoperative HE is the predictor of postoperative prolonged mechanical ventilation^26^. Prolonged postoperative mechanical ventilation could affect the blood flow and oxygenation of the graft^27,28^. Therefore, HE is an important feature of the proposed model.

The values of sodium and total bilirubin were serially collected from the preoperative phase to POD 7. Among these values, the sodium level at the end of surgery and total bilirubin level measured on POD 7 were selected as the resultant features. Elevated sodium levels may reflect the administration of large volumes of packed red blood cell, fresh frozen plasma, or albumin. Liver transplantation often requires massive transfusion of blood components. The sodium concentration in packed red blood cell is 150 mEq/L^29^ and that in fresh frozen plasma is 172 mEq/L^30^. Higher sodium concentration in these components can explain the higher sodium concentration at the end of surgery. Hypernatremia could also be associated with sodium bicarbonate infusion for correcting severe metabolic acidosis during surgery. Hyperbilirubinemia can be caused by impaired liver function, massive transfusions, or cholestasis^31,32^. Preoperative total bilirubin levels may be influenced by the preoperative status of recipients. Bilirubin levels before POD 7 could be affected by increased heme breakdown resulting from massive transfusion. Bilirubin levels measured on POD 7 may be associated with the postoperative graft function or cholestasis. Therefore, the total bilirubin level on POD 7 could be selected as an important predictive feature.

Thrombosis in the hepatic artery or portal vein is known to be a significant risk factor for graft survival in pediatric liver transplantation^7^. The incidence of early hepatic artery thrombosis is reported to be higher in children than in adults^33^. Hepatic artery and other vascular thromboses were reported as the most common cause of re-transplantation^1^. Early detection and revascularization can improve graft survival. Re-transplantation can be lifesaving if other interventions fail^34,35^. We used the thrombotic events observed until POD 7 as a feature, which was a relatively earlier period than that used in other studies^34–36^. A recent study reported a median time interval of 5.5 days between transplantation and hepatic artery complication^21^. Despite the different criteria, thrombosis in the hepatic artery still served as an important risk factor in graft survival.

### Limitation

First, we conducted a single-center retrospective study with a small sample size. Moreover, high correlations were observed between the collected features. To overcome the high dimensionality and collinearity in the dataset, we used a LASSO-based method to select predictive features. The top four predictive features were selected based on different clinical aspects and at different time points. We applied nested cross-validation during model development to avoid additional noise when splitting small-sized data into training and test datasets.

Second, we did not apply external validation to our prediction model because this was a single-center study. It is difficult to use serial laboratory data from other organizations for external validation. Further randomized controlled studies could help overcome this limitation and evaluate the impact of our machine learning model.

Third, we selected both living and deceased donor transplantations. Although not selected as a predictive feature, this might have biased the results of our study. In addition, the proportion of living donor transplantations was higher in our country, including our institution, than that in other countries. However, the inclusion of both types of donors could help in the generalizability of our study.

## Conclusion

We developed a machine learning model that predicts 90-days graft failure with high accuracy by overcoming the high dimensionality and collinearity of the dataset. The most predictive features were preoperative HE, Endop_Na, HA_thrombosis, and POD7_Tbilirubin. Based on this prediction model, we further developed a nomogram and risk scoring system for easy utilization in the routine clinical setting. These methods can serve as decision support systems for surgeons in identifying high-risk patients and preparing for proper intervention including re-transplantation during the early stage after surgery.

## Supplementary Information

Supplementary Figure S1.

Supplementary Figure S2.

Supplementary Figure S3.

Supplementary Figure S4.

Supplementary Table S1.

Supplementary Table S2.

Supplementary Table S3.

Supplementary Table S4.

Supplementary Table S5.

## Data Availability

The data is not publicly available due to privacy or ethical restrictions, but will be made available on reasonable request from the corresponding author, with the permission of the Institutional Review Board (IRB) of Severance Hospital. Restrictions apply to the availability of these data, which were used under license for this study. The code developed for this study is available on reasonable request from the corresponding author.

## References

[1] Elisofon SA (2020). Society of pediatric liver transplantation: Current registry status 2011–2018. Pediatr. Transplant..

[2] Cuenca AG, Kim HB, Vakili K (2017). Pediatric liver transplantation. Semin. Pediatr. Surg..

[3] Kwong AJ (2021). OPTN/SRTR 2019 annual data report: Liver. Am. J. Transpl. Off. J. Am. Soc. Transpl. Am. Soc. Transplant Surg..

[4] Kim WR (2019). OPTN/SRTR 2017 annual data report: Liver. Am. J. Transplant..

[5] Kohli R, Cortes M, Heaton ND, Dhawan A (2018). Liver transplantation in children: State of the art and future perspectives. Arch. Dis. Child..

[6] Tran LT, Carullo PC, Banh DPT, Vitu C, Davis PJ (2020). Pediatric liver transplantation: Then and now. J. Cardiothorac. Vasc. Anesth..

[7] McDiarmid SV, Anand R, Martz K, Millis MJ, Mazariegos G (2011). A multivariate analysis of pre-, peri-, and post-transplant factors affecting outcome after pediatric liver transplantation. Ann. Surg..

[8] Nacoti M (2011). Early detection of the graft failure after pediatric liver transplantation: A Bergamo experience. Acta Anaesthesiol. Scand..

[9] Ciria R (2012). Predictors of early graft survival after pediatric liver transplantation. Liver Transp. Off. Publ. Am. Assoc. Study Liver Dis. Int. Liver Transp. Soc..

[10] Wagener G, Raffel B, Young AT, Minhaz M, Emond J (2013). Predicting early allograft failure and mortality after liver transplantation: the role of the postoperative model for end-stage liver disease score. Liver Transpl. Off. Publ. Am. Assoc. Study Liver Dis. Int. Liver Transpl. Soc..

[11] Shelatkar T, Urvashi D, Shorfuzzaman M, Alsufyani A, Lakshmanna K (2022). Diagnosis of brain tumor using light weight deep learning model with fine-tuning approach. Comput. Math. Methods Med..

[12] Kumar V (2022). Addressing binary classification over class imbalanced clinical datasets using computationally intelligent techniques. Healthc. (Basel, Switz.).

[13] Lau L (2017). Machine-learning algorithms predict graft failure after liver transplantation. Transplantation.

[14] Dorado-Moreno M (2017). Dynamically weighted evolutionary ordinal neural network for solving an imbalanced liver transplantation problem. Artif. Intell. Med..

[15] Ayllón MD (2018). Validation of artificial neural networks as a methodology for donor-recipient matching for liver transplantation. Liver Transpl. Off. Publ. Am. Assoc. Study Liver Dis. Int. Liver Transpl. Soc..

[16] Tibshirani R (1996). Regression Shrinkage and Selection Via the Lasso. J. R. Stat. Soc. Ser. B (Methodol.).

[17] Zou H, Hastie T (2005). Regularization and variable selection via the elastic net. J. R. Stat. Soc. Ser. B (Stat. Methodol.).

[18] Meinshausen N, Bühlmann P (2010). Stability selection. J. R. Stat. Soc. Ser. B (Stat. Methodol.).

[19] Squires RH (2014). Evaluation of the pediatric patient for liver transplantation: 2014 practice guideline by the American Association for the Study of Liver Diseases, American Society of Transplantation and the North American Society for Pediatric Gastroenterology Hepatology and Nutrition. Hepatol. (Baltim. Md.).

[20] Loftus TJ (2020). Artificial Intelligence and Surgical Decision-making. JAMA Surg..

[21] Hong SK (2020). Outcomes of pediatric liver transplantation in Korea using two national registries. J. Clin. Med..

[22] Wadhwani SI (2019). Predicting ideal outcome after pediatric liver transplantation: An exploratory study using machine learning analyses to leverage Studies of Pediatric Liver Transplantation Data. Pediatr. Transplant..

[23] Vilstrup H (2014). Hepatic encephalopathy in chronic liver disease: 2014 Practice guideline by the American association for the study of liver diseases and the European association for the study of the liver. Hepatol. (Baltim. Md.).

[24] Wijdicks EF (2016). Hepatic encephalopathy. N. Engl. J. Med..

[25] Krishnarao A, Gordon FD (2020). Prognosis of hepatic encephalopathy. Clin. Liver Dis..

[26] Sahinturk H (2021). Risk factors for postoperative prolonged mechanical ventilation after pediatric liver transplantation. Exp. Clin. Transpl. Off. J. Middle East Soc. Organ Transpl..

[27] Yuan H (2014). Prognostic impact of mechanical ventilation after liver transplantation: A national database study. Am. J. Surg..

[28] Li J (2018). Immediate versus conventional postoperative tracheal extubation for enhanced recovery after liver transplantation: IPTE versus CTE for enhanced recovery after liver transplantation. Medicine.

[29] Ratcliffe JM, Elliott MJ, Wyse RK, Hunter S, Alberti KG (1986). The metabolic load of stored blood. Implications for major transfusions in infants. Arch. Dis. Child..

[30] Ewalenko P, Deloof T, Peeters J (1986). Composition of fresh frozen plasma. Crit. Care Med..

[31] Sticova E, Jirsa M (2013). New insights in bilirubin metabolism and their clinical implications. World J. Gastroenterol..

[32] Muniyappa P, Kelley D (2020). Hyperbilirubinemia in pediatrics: Evaluation and care. Curr. Probl. Pediatr. Adolesc. Health Care.

[33] Bekker J, Ploem S, De Jong KP (2009). Early hepatic artery thrombosis after liver transplantation: A systematic review of the incidence, outcome and risk factors. Am. J. Transplant..

[34] Sevmis S (2011). Management of early hepatic arterial thrombosis after pediatric living-donor liver transplantation. Transpl. Proc..

[35] Kutluturk K (2019). Early hepatic artery thrombosis after pediatric living donor liver transplantation. Transpl. Proc..

[36] Bezinover D (2019). Perioperative thrombotic complications associated with pediatric liver transplantation: A UNOS database evaluation. HPB (Oxf.).

