# Supplementary figures and images for: Predicting graft failure in pediatric liver transplantation based on early biomarkers using machine learning models
Source: Sci Rep. 2022 Dec 27;12:22411. doi: 10.1038/s41598-022-25900-0 (PMC9794703; doi:10.1038/s41598-022-25900-0)

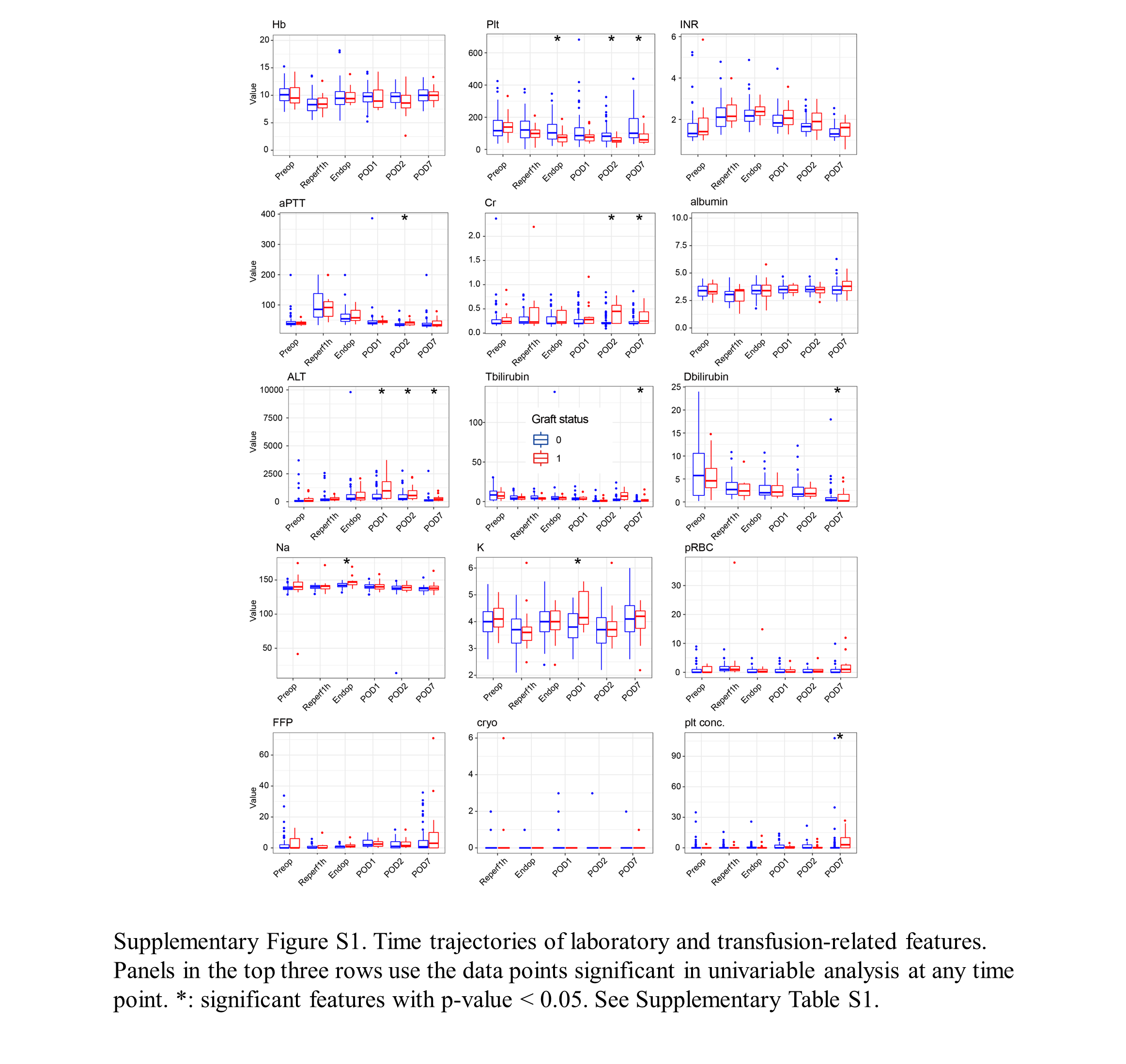

Supplement: Supplementary file 1 — Supplementary Figure S1. [file 41598_2022_25900_MOESM1_ESM.tif]

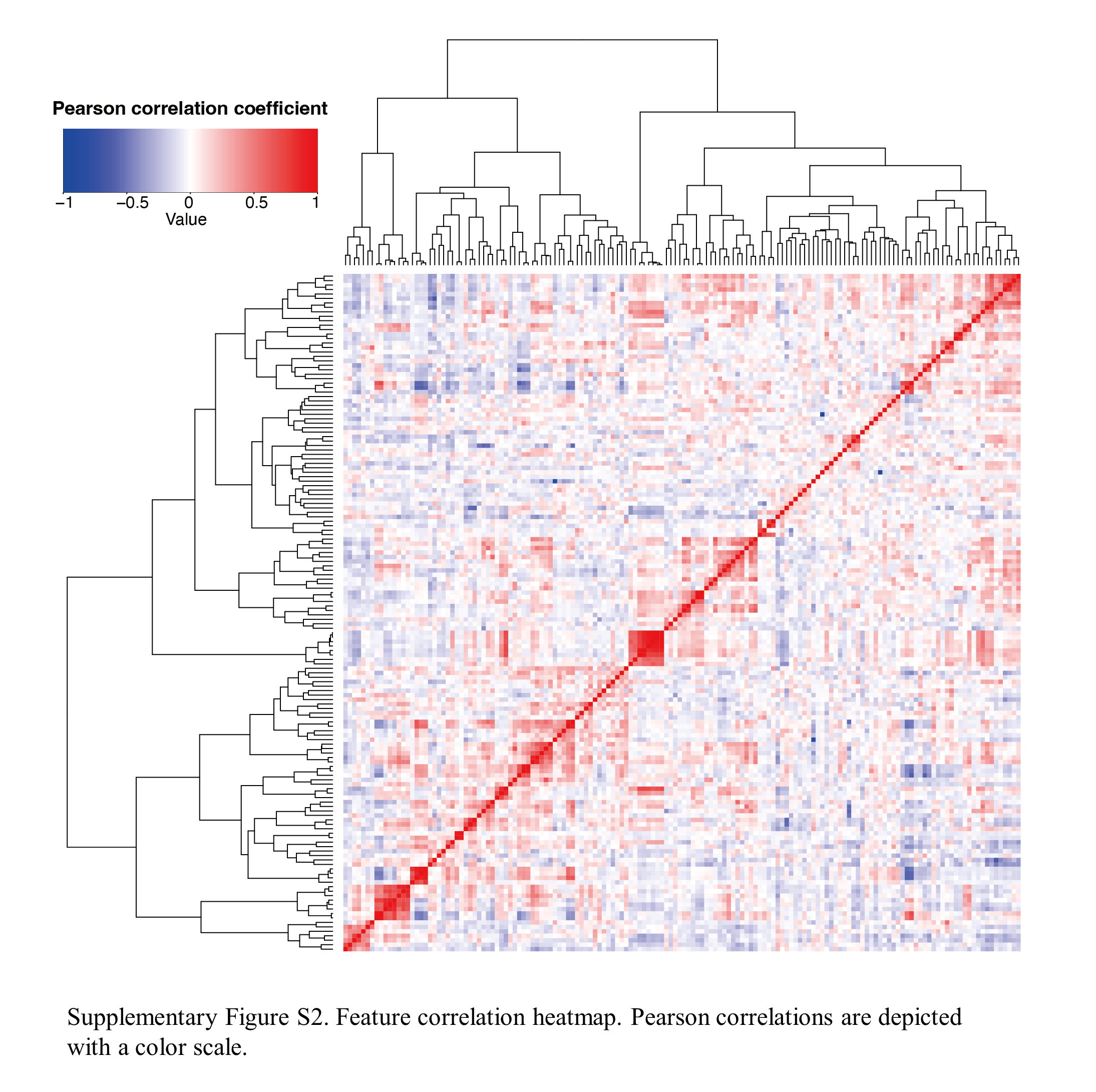

Supplement: Supplementary file 2 — Supplementary Figure S2. [file 41598_2022_25900_MOESM2_ESM.tif]

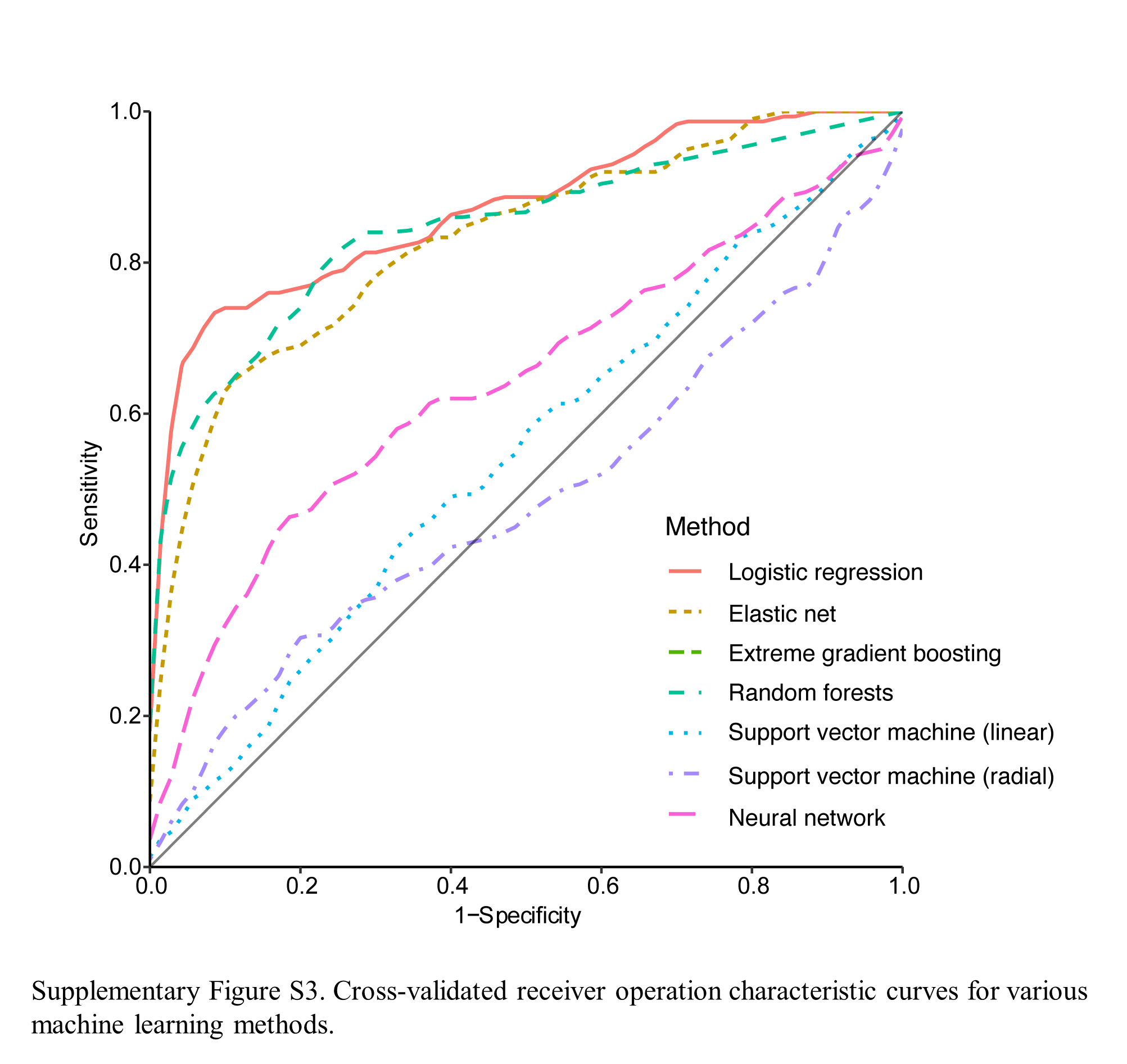

Supplement: Supplementary file 3 — Supplementary Figure S3. [file 41598_2022_25900_MOESM3_ESM.tif]

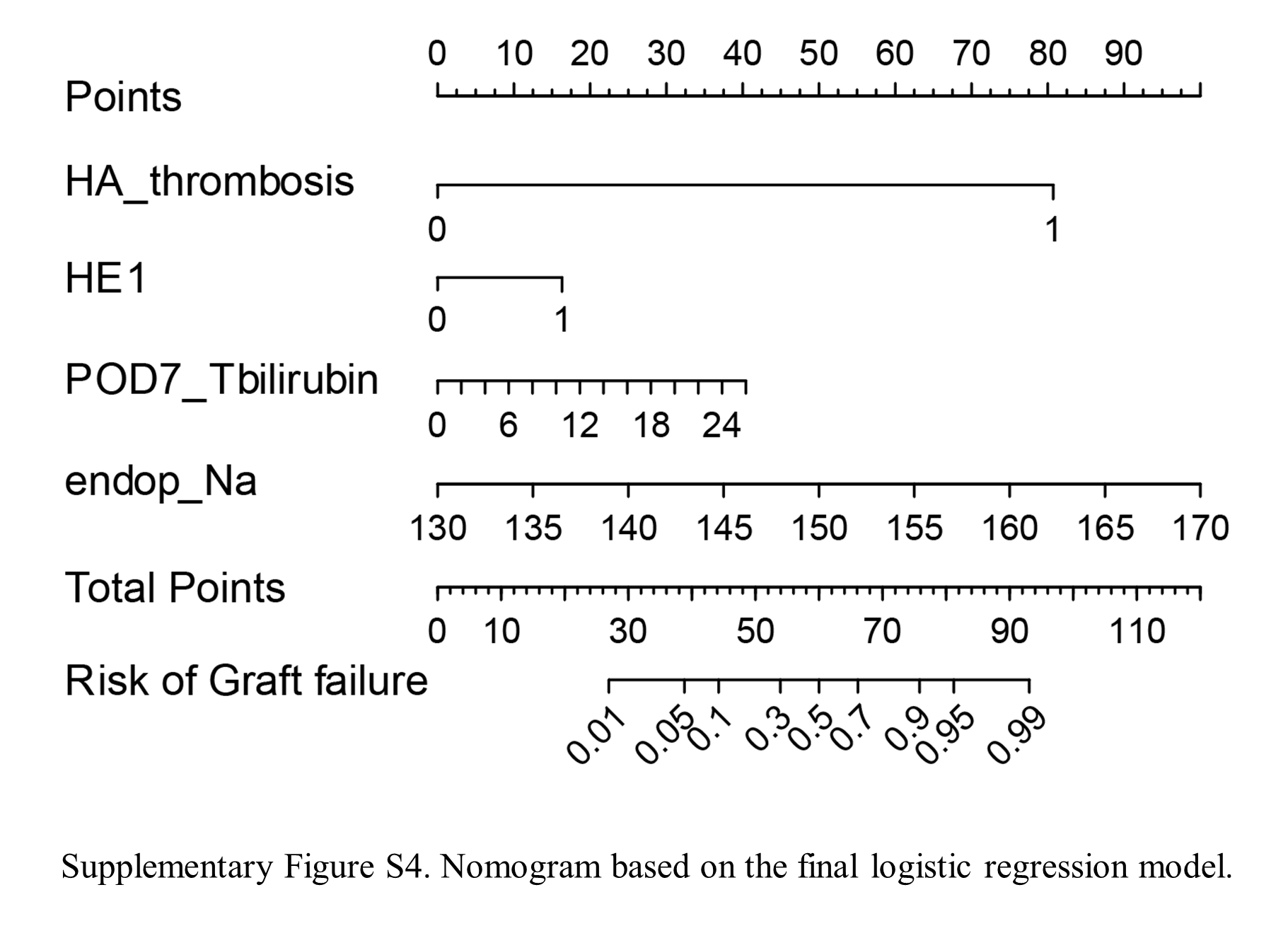

Supplement: Supplementary file 4 — Supplementary Figure S4. [file 41598_2022_25900_MOESM4_ESM.tif]
