# Supplementary material for: Predicting graft failure in pediatric liver transplantation based on early biomarkers using machine learning models
Source: Sci Rep. 2022 Dec 27;12:22411. doi: 10.1038/s41598-022-25900-0 (PMC9794703; doi:10.1038/s41598-022-25900-0)
Supplement: Supplementary file 5 — Supplementary Table S1. [file 41598_2022_25900_MOESM5_ESM.docx]

| **Title** | **Outcomes of interest** | **Main outcomes** | **Exclusion criteria** |
| --- | --- | --- | --- |
| Machine-Learning Algorithms Predict Graft Failure After Liver Transplantation^1^ | Graft failure at 30 days and 3 months | The models predict graft survival with an AUROC of 0.818 at 30 days and 0.715 at 3 months. | Pediatric and living donor transplantations were excluded. |
| Dynamically weighted evolutionary ordinal neural network for solving an imbalanced liver transplantation problem^2^ | Graft survival at less than 15 days, between 15 and 90 days, between 90 and 365 days and more than 365 days. | The model correctly predicted more than 73% of the transplantation results, with a geometric mean of the sensitivities of 31.46% | Patients undergoing partial, split or living-donor liver transplantation were excluded. |
| Validation of Artificial Neural Networks as a Methodology for Donor-Recipient Matching for Liver Transplantation^3^ | Graft survival at 3 and 12 months | The models predict graft survival with AUROC of 0.94, MS-AUROC of 0.94 at 3 months and AUROC of 0.78, MS-AUROC of 0.82 at 12 months. | Pediatric, living donor transplantations, and hepatocellular carcinoma patients were excluded. |

Supplementary Table S1. Machine learning models predicting graft survival/failure in liver transplantation.

Abbreviations: AUROC: area under receiver operation characteristic curve, MS: minimum sensitivity.

**References**

1. Lau, L. *et al.* Machine-Learning Algorithms Predict Graft Failure After Liver Transplantation. *Transplantation* **101**, e125-e132. https://doi.org/10.1097/tp.0000000000001600 (2017).

2. Dorado-Moreno, M. *et al.* Dynamically weighted evolutionary ordinal neural network for solving an imbalanced liver transplantation problem. *Artificial intelligence in medicine* **77**, 1-11. https://doi.org/10.1016/j.artmed.2017.02.004 (2017).

3. Ayllón, M. D. *et al.* Validation of artificial neural networks as a methodology for donor-recipient matching for liver transplantation. *Liver transplantation : official publication of the American Association for the Study of Liver Diseases and the International Liver Transplantation Society* **24**, 192-203. https://doi.org/10.1002/lt.24870 (2018).
