# Supplementary material for: Predicting graft failure in pediatric liver transplantation based on early biomarkers using machine learning models
Source: Sci Rep. 2022 Dec 27;12:22411. doi: 10.1038/s41598-022-25900-0 (PMC9794703; doi:10.1038/s41598-022-25900-0)
Supplement: Supplementary file 6 — Supplementary Table S2. [file 41598_2022_25900_MOESM6_ESM.docx]

Supplementary Table S2. Abbreviations and descriptions.

| **Abbreviation** | **Description** |
| --- | --- |
| LT | Liver transplantation |
| age_mon | Age (month) |
| optime | Operation time |
| anetime | Anesthesia time |
| UO  pRBC  FFP  Cryo  Plt conc  INR  aPTT  Cr  ALT  CRP  CRRT  POD | Urine output  Packed red blood cell  Fresh frozen plasma  Cryoprecipitate  Platelet concentrate  International Normalized normalized Ratioratio  Activated Ppartial Thromboplastin thromboplastin Timetime  Creatinine  Alanine transaminase  C-reactive protein  Continuous renal replacement therapy  Post operative day |
| Intraop_pRBC | Total pRBC transfusion amount during operation |
| Intraop_FFP | Total FFP transfusion amount during operation |
| Intraop_Cryo | Total cryo transfusion amount during operation |
| Intraop_plt conc. | Total plt conc concentrated transfusion amount during operation |
| Crystalloid | Crystalloid amount during operation |
| preop_Hb | Preoperative hemoglobin level |
| preop_Plt | Preoperative platelet count |
| preop_ INR | Preoperative INR |
| preop_aPTT | Preoperative aPTT |
| preop_Cr | Preoperative Cr level |
| preop_albumin | Preoperative albumin level |
| preop_ALT | Preoperative ALT level |
| preop_Tbilirubin | Preoperative total bilirubin level |
| preop_Dbilirubin | Preoperative direct bilirubin level |
| preop_Na | Preoperative Na level |
| preop_K | Preoperative K level |
| preop_CRP | Preoperative CRP level |
| preop_PRBC | Preoperative pRBC transfusion amount |
| preop_FFP | Preoperative FFP transfusion amount |
| preop_plt conc. | Preoperative plt conc transfusion amount |
| reperf1h_Hb | Hemoglobin level 1 hour h after reperfusion |
| reperf1h_Plt | Platelet count 1 hour h after reperfusion |
| reperf1h_INR | INR 1 hour h after reperfusion |
| reperf1h_aPTT | aPTT 1 hour h after reperfusion |
| reperf1h_Cr | Cr level 1 hour h after reperfusion |
| reperf1h_albumin | Albumin 1 hour h after reperfusion |
| reperf1h_ALT | ALT 1 hour h after reperfusion |
| reperf1H_Tbilirubin | Total bilirubin 1 hour h after reperfusion |
| reperf1h_Na | Na level 1 hour h after reperfusion |
| reperf1h_K | K level 1 hour h after reperfusion |
| reperf1h_pRBC | Intraoperative pRBC transfusion amount until 1 hour h after reperfusion |
| reperf1h_FFP | Intraoperative FFP transfusion amount until 1 hour h after reperfusion |
| reperf1h_cryo | Intraoperative cryo transfusion amount until 1 hour h after reperfusion |
| reperf1h_plt conc. | Plt conc transfusion until 1 hour h after reperfusion |
| endop_Hb | Hemoglobin level at the end of operation |
| endop_Plt | Platelet count at the end of operation |
| endop_INR | INR at the end of operation |
| endop_aPTT | aPTT at the end of operation |
| endop_Cr | Cr level at the end of operation |
| endop_albumin | Albumin level at the end of operation |
| endop_ALT | ALT at the end of operation |
| endop_Tbilirubin | Total bilirubin level at the end of operation |
| endop_Dbilirubin | Direct bilirubin level at the end of operation |
| endop_Na | Na level at the end of operation |
| endop_K | K level at the end of operation |
| endop_pRBC | pRBC transfusion amount from 1 hour h after reperfusion to the end of operation |
| endop_FFP | FFP transfusion amount from 1 hour h after reperfusion to the end of operation |
| endop_cryo | Cryo transfusion amount from 1 hour h after reperfusion to the end of operation |
| endop_ plt conc. | Plt conc transfusion amount from 1 hour h after reperfusion to the end of operation |
| POD1_Hb | Hemoglobin level at on POD 1 |
| POD1_Plt | Platelet count at on POD 1 |
| POD1_INR | INR at on POD 1 |
| POD1_aPTT | aPTT at on POD 1 |
| POD1_Cr | Cr level at on POD 1 |
| POD1_albumin | Albumin level at on POD 1 |
| POD1_ALT | ALT level at on POD 1 |
| POD1_Tbilirubin | Total bilirubin level at on POD 1 |
| POD1/Preop_Tbilirubin | Ratio of total bilirubin level between at on POD 1 and at preoperative period |
| POD1_Dbilirubin | Direct bilirubin level at on POD 1 |
| POD1/Preop_Dbilirubin | Ratio of direct bilirubin level between at on POD 1 and at preoperative period |
| POD1_Na | Na level at on POD 1 |
| POD1_K | K level at on POD 1 |
| POD1_pRBC | pRBC transfusion amount at on POD 1 |
| POD1_FFP | FFP transfusion amount at on POD 1 |
| POD1_cryo | Cryo transfusion amount at on POD 1 |
| POD1_ plt conc. | Plt conc transfusion amount at on POD 1 |
| POD2_Hb | Hemoglobin level at on POD 2 |
| POD2_Plt | Platelet count on at POD 2 |
| POD2_INR | INR at on POD 2 |
| POD2_aPTT | aPTT at on POD 2 |
| POD2_Cr | Cr level at on POD 2 |
| POD2_albumin | Albumin level at on POD 2 |
| POD2_ALT | ALT level at on POD 2 |
| POD2_Tbilirubin | Total bilirubin level at on POD 2 |
| POD2/Preop_Tbilirubin | Ratio of total bilirubin level between at POD 2 and at preoperative period |
| POD2_Dbilirubin | Direct bilirubin level at on POD 2 |
| POD2/Preop_Dbilirubin | Ratio of direct bilirubin level between at POD 2 and at preoperative period |
| POD2_Na | Na level at on POD 2 |
| POD2_K | K level at on POD 2 |
| POD2_pRBC | pRBC transfusion amount at on POD 2 |
| POD2_FFP | FFP transfusion amount at on POD 2 |
| POD2_cryo | Cryo transfusion amount at on POD 2 |
| POD2_ plt conc. | Plt conc transfusion amount at on POD 2 |
| POD7_Hb | Hemoglobin level at on POD 7 |
| POD7_Plt | Platelet count at on POD 7 |
| POD7_INR | INR at on POD 7 |
| POD7_aPTT | aPTT at on POD 7 |
| POD7_Cr | Cr level at on POD 7 |
| POD7_albumin | Albumin level at on POD 7 |
| POD7_ALT | ALT level at on POD 7 |
| POD7_Tbilirubin | Total bilirubin level at POD 7 |
| POD7/Preop_Tbilirubin | Ratio of total bilirubin level between at POD 7 and at preoperative period |
| POD7_Dbilirubin | Direct bilirubin level at on POD 7 |
| POD7/Preop_Dbilirubin | Ratio of direct bilirubin level between at POD 7 and at preoperative period |
| POD7_Na | Na level at on POD 7 |
| POD7_K | K level at on POD 7 |
| POD7_pRBC | pRBC transfusion amount at on POD 7 |
| POD7_FFP | FFP transfusion amount at on POD 7 |
| POD7_cryo | Cryo transfusion amount at on POD 7 |
| POD7_ plt conc. | Plt conc transfusion amount at on POD 7 |
| ASA | American Society of Anesthesiology |
| elective | Elective/emergency operation |
| HA_thrombosis | Hepatic artery thrombosis until POD 7 |
| HV_thrombosis | Hepatic vein thrombosis until POD 7 |
| PV_thrombosis | Portal vein thrombosis until POD 7 |
| Cx_bile duct | Bile duct complication until POD 7 |
| Cx_infection | Infection complication until POD 7 |
| gstatus | Graft status at 90 days after transplantation |
| prev_op | Previous surgical history before liver transplant surgery |
| ascites | Preoperative ascites |
| HE | Preoperative hepatic encephalopathy |
| EV | Preoperative esophageal varix |
| splenomegaly | Preoperative splenomegaly |
| abnl echo | Preoperative abnormal echocardiography |
| periop_CRRT | Perioperative CRRT |
| Preop_CRRT | Preoperative CRRT |
| Intra_CRRT | Intraoperative CRRT |
| postop_CRRT | Postoperative CRRT |
| Preop_ICU | Preoperative intensive care unit admission |
| Preop_vent | Preoperative mechanical ventilation |
| Reperfusion time | Time from induction of anesthesia to reperfusion |
| Anhepatic phase | Duration of anhepatic phase |
| intraop_vaso | Intraoperative vasopressor use |
| ABO_m | ABO mismatch between donor and recipient |
| sex_d | Donor sex |
| age_d | Donor age |
| CV | Cross validation |
| ML | Machine learning |
| LR | Logistic regression |
| AUROC | Area under receiver operation characteristic curve |
| AUPR | Area under precision-recall curve |
|  |  |
