# Supplementary material for: Predicting graft failure in pediatric liver transplantation based on early biomarkers using machine learning models
Source: Sci Rep. 2022 Dec 27;12:22411. doi: 10.1038/s41598-022-25900-0 (PMC9794703; doi:10.1038/s41598-022-25900-0)
Supplement: Supplementary file 7 — Supplementary Table S3. [file 41598_2022_25900_MOESM7_ESM.docx]

Supplementary Table S3. Covariates measured at multiple time points.

|  | All | No graft failure | Graft failure | p-value |
| --- | --- | --- | --- | --- |
|  | N = 87 | N = 70 (80.5%) | N = 17 (19.5%) |  |
| Intraop_pRBC | 2.5 (±5.8) | 1.9 (±1.9) | 5.0 (±12.4) | 0.323 |
| Intraop_FFP | 1.6 (±2.2) | 1.4 (±1.5) | 2.3 (±3.9) | 0.277 |
| Intraop_Cryo | 0.4 (±1.6) | 0.3 (±1.0) | 0.8 (±2.9) | 0.816 |
| Intraop_plt conc. | 1.4 (±3.1) | 1.3 (±2.6) | 1.8 (±4.5) | 0.912 |
| Crystalloid (mL/kg/h) | 13.5 (±6.9) | 12.5 (±4.7) | 17.3 (±11.8) | 0.100 |
| preop_Hb | 10.2 (±1.6) | 10.2 (±1.6) | 10.0 (±1.8) | 0.725 |
| preop_Plt | 142.0 (±81.8) | 141.3 (±83.9) | 144.2 (±75.1) | 0.567 |
| preop_INR | 1.7 (±0.9) | 1.7 (±0.8) | 1.8 (±1.1) | 0.507 |
| preop_aPTT | 43.5 (±20.5) | 44.2 (±22.6) | 40.6 (±7.5) | 0.902 |
| preop_Cr | 0.31 (±0.27) | 0.30 (±0.29) | 0.31 (±0.18) | 0.070 |
| preop_albumin | 3.4 (±0.5) | 3.4 (±0.5) | 3.5 (±0.6) | 0.489 |
| preop_ALT | 208.0 (±529.0) | 202.4 (±569.3) | 229.2 (±321.2) | 0.346 |
| preop_Tbilirubin | 9.3 (±7.8) | 9.4 (±8.2) | 8.4 (±5.6) | 0.910 |
| preop_Dbilirubin | 6.6 (±5.8) | 6.8 (±6.1) | 5.6 (±4.3) | 0.760 |
| preop_Na | 138.0 (±12.1) | 138.6 (±3.9) | 137.8 (±27.0) | 0.291 |
| preop_K | 4.0 (±0.5) | 4.0 (±0.6) | 4.1 (±0.5) | 0.405 |
| preop_CRP | 14.7 (±23.2) | 16.4 (±25.4) | 7.7 (±7.6) | 0.371 |
| Preop_NH3 | 108.0 (±53.2) | 104.4 (±46.7) | 120.9 (±74.4) | 0.649 |
| preop_PRBC | 1.0 (±1.7) | 1.0 (±1.8) | 0.8 (±1.1) | 0.933 |
| preop_FFP | 2.6 (±5.6) | 2.5 (±5.9) | 3.0 (±4.3) | 0.317 |
| preop_ plt conc | 1.3 (±4.9) | 1.5 (±5.4) | 0.2 (±1.0) | 0.251 |
| reperf1h_Hb | 8.5 (±1.7) | 8.4 (±1.7) | 8.6 (±1.6) | 0.684 |
| reperf1h_Plt | 127.0 (±76.1) | 133.8 (±79.9) | 100.9 (±51.2) | 0.190 |
| reperf1h_INR | 2.3 (±0.7) | 2.2 (±0.7) | 2.3 (±0.6) | 0.512 |
| reperf1h_aPTT | 106.0 (±57.0) | 106.5 (±58.4) | 102.2 (±52.5) | 0.983 |
| reperf1h_Cr | 0.32 (±0.26) | 0.29 (±0.14) | 0.46 (±0.53) | 0.537 |
| reperf1h_albumin | 3.0 (±0.7) | 3.0 (±0.7) | 3.0 (±0.8) | 0.867 |
| reperf1h_ALT | 285.0 (±443.0) | 284.3 (±479.5) | 285.6 (±207.4) | 0.095 |
| reperf1H_Tbilirubin | 5.3 (±3.4) | 5.3 (±3.6) | 5.0 (±2.5) | 0.901 |
| reperf1h_Na | 140.0 (±5.1) | 139.7 (±3.8) | 141.3 (±8.9) | 0.881 |
| reperf1h_K | 3.7 (±0.7) | 3.7 (±0.6) | 3.7 (±0.8) | 0.611 |
| reperf1h_pRBC | 1.7 (±4.1) | 1.3 (±1.3) | 3.4 (±9.0) | 0.688 |
| reperf1h_FFP | 0.8 (±1.5) | 0.8 (±1.2) | 1.1 (±2.4) | 0.814 |
| reperf1h_cryo | 0.14 (±0.72) | 0.07 (±0.35) | 0.41 (±1.46) | 0.238 |
| reperf1h_ plt conc | 0.74 (±2.24) | 0.79 (±2.38) | 0.53 (±1.6) | 0.627 |
| endop_Hb | 9.7 (±2.1) | 9.6 (±2.2) | 9.9 (±1.5) | 0.563 |
| endop_Plt | 110.0 (±68.7) | 117.8 (±71.6) | 77.4 (±43.0) | 0.033 |
| endop_INR | 2.3 (±0.6) | 2.3 (±0.6) | 2.4 (±0.4) | 0.156 |
| endop_aPTT | 61.9 (±25.3) | 60.9 (±25.9) | 65.9 (±23.2) | 0.294 |
| endop_Cr | 0.29 (±0.14) | 0.29 (±0.14) | 0.31 (±0.16) | 0.616 |
| endop_albumin | 3.7 (±3.2) | 3.8 (±3.5) | 3.5 (±1.0) | 0.906 |
| endop_ALT | 609.0 (±1110.0) | 607.6 (±1202.0) | 615.1 (±650.9) | 0.902 |
| endop_Tbilirubin | 5.0 (±3.1) | 5.2 (±3.2) | 4.1 (±2.3) | 0.294 |
| endop_Dbilirubin | 3.1 (±2.2) | 3.3 (±2.2) | 2.7 (±2.0) | 0.338 |
| endop_Na | 143.0 (±4.8) | 142.3 (±3.3) | 146.4 (±8.0) | 0.016 |
| endop_K | 4.0 (±0.6) | 4.0 (±0.6) | 4.0 (±0.6) | 0.984 |
| endop_pRBC | 0.6 (±1.7) | 0.5 (±0.8) | 1.4 (±3.6) | 0.193 |
| endop_FFP | 1.1 (±1.2) | 1.0 (±1.0) | 1.4 (±1.7) | 0.250 |
| endop_cryo | 0.02 (±0.15) | 0.03 (±0.17) | 0 (±0) | >0.999 |
| endop_ plt conc | 1.0 (±3.3) | 1.0 (±3.3) | 1.2 (±3.2) | 0.955 |
| POD1_Hb | 9.6 (±1.8) | 9.7 (±1.6) | 9.9 (±2.3) | 0.610 |
| POD1_Plt | 111.0 (±96.3) | 117.4 (±104.2) | 83.2 (±40.4) | 0.287 |
| POD1_INR | 2.0 (±0.5) | 2.0 (±0.5) | 2.2 (±0.6) | 0.192 |
| POD1_aPTT | 47.6 (±38.2) | 48.1 (±42.3) | 45.8 (±7.6) | 0.148 |
| POD1_Cr | 0.3 (±0.2) | 0.3 (±0.2) | 0.4 (±0.3) | 0.055 |
| POD1_albumin | 3.5 (±0.5) | 3.5 (±0.5) | 3.5 (±0.4) | 0.957 |
| POD1_ALT | 708.0 (±747.0) | 587.9 (±610.8) | 1235.0 (±1041.0) | 0.005 |
| POD1_Tbilirubin | 6.3 (±14.8) | 6.7 (±16.3) | 4.5 (±2.3) | 0.894 |
| POD1.Preop_Tbilirubin | 2.0 (±5.5) | 2.2 (±6.1) | 1.3 (±1.8) | 0.748 |
| POD1_Dbilirubin | 2.7 (±1.9) | 2.8 (±2.0) | 2.5 (±1.5) | 0.870 |
| POD1.Preop_Tbilirubin | 1.4 (±2.4) | 1.5 (±2.5) | 1.2 (±1.7) | 0.657 |
| POD1_Na | 140.0 (±5.0) | 140.1 (±4.4) | 141.3 (±7.1) | 0.504 |
| POD1_K | 4.0 (±0.6) | 3.9 (±0.6) | 4.4 (±0.7) | 0.004 |
| POD1_pRBC | 0.7 (±1.2) | 0.7 (±1.2) | 0.7 (±1.2) | 0.909 |
| POD1_FFP | 2.8 (±2.5) | 2.8 (±2.6) | 2.7 (±2.1) | >0.999 |
| POD1_cryo | 0.08 (±0.41) | 0.10 (±0.46) | 0 (±0) | >0.999 |
| POD1_ plt conc | 1.6 (±3.1) | 1.7 (±3.4) | 0.8 (±1.5) | 0.814 |
| POD2_Hb | 9.5 (±1.6) | 9.7 (±1.2) | 8.9 (±2.7) | 0.271 |
| POD2_Plt | 85.9 (±56.1) | 92.0 (±59.5) | 58.9 (±25.3) | 0.028 |
| POD2_INR | 1.7 (±0.4) | 1.7 (±0.4) | 1.9 (±0.6) | 0.109 |
| POD2_aPTT | 37.5 (±8.6) | 36.7 (±8.4) | 41.5 (±9.0) | 0.023 |
| POD2_Cr | 0.28 (±0.17) | 0.25 (±0.14) | 0.42 (±0.21) | <0.001 |
| POD2_albumin | 4.9 (±13.0) | 5.2 (±14.3) | 3.4 (±0.5) | 0.742 |
| POD2_ALT | 569.0 (±583.0) | 519.7 (±550.5) | 799.3 (±690.6) | 0.041 |
| POD2_Tbilirubin | 4.2 (±3.1) | 4.2 (±3.2) | 4.4 (±2.9) | 0.751 |
| POD2.Preop_Tbilirubin | 1.3 (±2.1) | 1.3 (±2.1) | 1.5 (±2.3) | 0.982 |
| POD2_Dbilirubin | 2.4 (±2.1) | 2.5 (±2.2) | 2.1 (±1.2) | 0.986 |
| POD2.Preop_Tbilirubin | 1.2 (±2.3) | 1.2 (±2.4) | 1.3 (±1.7) | 0.770 |
| POD2_Na | 137.0 (±14.1) | 136.9 (±15.4) | 139.4 (±5.3) | 0.746 |
| POD2_K | 3.7 (±0.6) | 3.6 (±0.6) | 3.9 (±0.8) | 0.548 |
| POD2_pRBC | 0.4 (±0.8) | 0.4 (±0.6) | 0.7 (±1.2) | 0.138 |
| POD2_FFP | 2.5 (±2.9) | 2.5 (±2.7) | 2.8 (±3.5) | 0.744 |
| POD2_cryo | 0.03 (±0.3) | 0.04 (±0.36) | 0 (±0) | >0.999 |
| POD2_ plt conc | 1.6 (±3.2) | 1.7 (±3.3) | 1.3 (±2.6) | 0.480 |
| POD7_Hb | 11.4 (±12.1) | 11.6 (±13.3) | 10.1 (±1.4) | 0.908 |
| POD7_Plt | 128.0 (±87.8) | 137.4 (±91.1) | 81.8 (±51.0) | 0.006 |
| POD7_INR | 1.4 (±0.3) | 1.4 (±0.3) | 1.5 (±0.5) | 0.174 |
| POD7_aPTT | 39.2 (±21.2) | 38.9 (±22.3) | 40.8 (±15.8) | 0.632 |
| POD7_Cr | 0.27 (±0.14) | 0.25 (±0.13) | 0.34 (±0.16) | 0.012 |
| POD7_albumin | 3.6 (±0.7) | 3.5 (±0.6) | 3.8 (±0.7) | 0.102 |
| POD7_ALT | 201.0 (±331.0) | 177.5 (±337.6) | 313.2 (±278.7) | 0.008 |
| POD7_Tbilirubin | 3.9 (±4.6) | 3.0 (±3.7) | 8.0 (±6.1) | <0.001 |
| POD7.Preop_Tbilirubin | 1.2 (±2.4) | 0.9 (±1.6) | 2.7 (±4.3) | 0.016 |
| POD7_Dbilirubin | 2.3 (±2.7) | 2.0 (±2.5) | 3.6 (±3.0) | 0.012 |
| POD7.Preop_Dbilirubin | 1.3 (±2.7) | 1.1 (±2.4) | 2.4 (±3.5) | 0.124 |
| POD7_Na | 138.0 (±4.7) | 137.1 (±3.5) | 139.6 (±8.2) | 0.288 |
| POD7_K | 4.1 (±0.7) | 4.1 (±0.7) | 3.9 (±0.7) | 0.329 |
| POD7_pRBC | 1.1 (±2.2) | 0.7 (±1.5) | 2.6 (±3.7) | 0.053 |
| POD7_FFP | 5.7 (±11.3) | 4.6 (±8.4) | 10.4 (±19.6) | 0.497 |
| POD7_cryo | 0.04 (±0.24) | 0.03 (±0.24) | 0.07 (±0.26) | 0.245 |
| POD7_ plt conc | 3.8 (±13.2) | 3.2 (±13.9) | 6.8 (±8.9) | <0.001 |

The numbers denote the mean (±SD).
