# Supplementary material for: Predicting graft failure in pediatric liver transplantation based on early biomarkers using machine learning models
Source: Sci Rep. 2022 Dec 27;12:22411. doi: 10.1038/s41598-022-25900-0 (PMC9794703; doi:10.1038/s41598-022-25900-0)
Supplement: Supplementary file 8 — Supplementary Table S4. [file 41598_2022_25900_MOESM8_ESM.docx]

Supplementary Table S4. Comparison of model performance across various machine learning models.

| Methods | AUROC | AUPR |
| --- | --- | --- |
| Logistic regression | 0.898 | 0.882 |
| Elastic net | 0.898 | 0.852 |
| RF | 0.861 | 0.855 |
| XGB | 0.821 | 0.812 |
| SVM (linear) | 0.539 | 0.506 |
| SVM (radial) | 0.692 | 0.679 |
| Neural network | 0.732 | 0.656 |

Abbreviations: XGB, extreme gradient boosting; RF, random forests; SVM, support vector machine.
