# Supplementary material for: Predicting graft failure in pediatric liver transplantation based on early biomarkers using machine learning models
Source: Sci Rep. 2022 Dec 27;12:22411. doi: 10.1038/s41598-022-25900-0 (PMC9794703; doi:10.1038/s41598-022-25900-0)
Supplement: Supplementary file 9 — Supplementary Table S5. [file 41598_2022_25900_MOESM9_ESM.docx]

Supplementary Table S5. Final logistic regression model.

|  | Coefficient | OR | 95% CI | P-value |
| --- | --- | --- | --- | --- |
| (Intercept) | -53.5 | 5.9$\times$10^-24^ | [6.5$\times$10^-91^–3.2$\times$10^-11^] | 0.002 |
| HA_thrombosis | 21.1 | 1.4$\times$10^9^ | [2.2$\times$10^6^–4.9$\times$10^14^] | 0.005 |
| HE | 2.3 | 9.6 | [9.0$\times$10^-1^–1.9$\times$10^9^] | 0.055 |
| POD7_Tbilirubin (per 10 mg/dL) | 2.2 | 8.7 | [2.2–3.0$\times$10^4^] | 0.017 |
| endop_Na (per 10mmol/L) | 3.5 | 3.2$\times$10^1^ | [4.1–8.1$\times$10^5^] | 0.004 |
